# Return to work status of patients under 65 years of age with osteonecrosis of the femoral head after total hip arthroplasty

**DOI:** 10.1186/s13018-023-04283-6

**Published:** 2023-10-18

**Authors:** Mengfei Wang, Rushun Zhao, Yangquan Hao, Peng Xu, Chao Lu

**Affiliations:** 1 Department of Joint Surgery, Xi’an Hong Hui Hospital, Xi’an Jiaotong University Health Science Center, No. Youyi East Road, Nanshaomen, Xi’an, 710054 Shaanxi Province People’s Republic of China; 2 Shaanxi University of Traditional Chinese Medicine, Xi’an, 712046 Shaanxi Province People’s Republic of China

**Keywords:** Arthroplasty, Hip, Osteonecrosis of the femoral head (ONFH), Return, Work

## Abstract

**Objective:**

This aimed to evaluate the status of return to work (RTW) in patients with osteonecrosis of the femoral head (ONFH) after total hip arthroplasty (THA).

**Methods:**

The baseline characteristics of all patients in this retrospective study were obtained from the hospital patient database. The relevant changes in patients' working conditions, as well as the numerical rating scale (NRS), Harris Hip Score (HHS), self-assessment of work ability, and Likert scale satisfaction assessment were obtained through video call follow-ups.

**Results:**

118 patients (response rate: 83%) were ultimately included in this study. The average length of time for the patients to stop working preoperatively was 20.7 weeks. Ninety-four patients (24 women and 70 men) who underwent THA had RTW status, with a mean RTW time of 21.0 weeks. Men had a significantly higher proportion of final RTW and a significantly faster RTW than women. Significant differences in smoking, drinking, cardiovascular diseases, changes in working levels, variations in the types of physical work, changes in working hours, and pain symptoms were observed between the RTW and Non-RTW populations. The patients with a positive RTW status had higher postoperative HHS scores, lower postoperative NRS scores, and higher self-assessment of work ability than patients who had a negative RTW status.

**Conclusion:**

Ultimately, 80% of patients achieved RTW status. Drinking, sex, change in working level, variation in the type of physical work, change in working hours, post-surgery HHS score and self-assessment of work ability can serve as predictive factors for RTW.

## Introduction

Osteonecrosis of the femoral head (ONFH) is a disease in which the blood supply system of the femoral head is damaged. Osteocyte apoptosis is present, and structural changes of the femoral head occur, ultimately resulting in the collapse of the femoral head and clinical symptoms such as joint pain and dysfunction [1]. ONFH can be classified as traumatic femoral head necrosis or non-traumatic femoral head necrosis [2]. The etiology of non-traumatic ONFH is complex, and the two most common risk factors are steroid use and excessive alcohol consumption [3, 4]. The etiology of traumatic ONFH is more readily understood. Relevant studies have reported over 20 million cases of confirmed ONFH worldwide [5], with China recording approximately 8 million cases of confirmed ONFH, amounting to 100,000–200,000 cases/year [1, 6].

Regardless of the cause for ONFH, once the disease progresses to subchondral fracture, femoral head collapse, and painful osteoarthritis, total hip arthroplasty (THA) has advantages such as better functional outcomes and lower risk of revision surgery, and is usually recommended as the primary treatment method [7–10]. Nipun et al. [11] found that the prevalence of THA treatment for ONFH in the United States was extremely high between 2009 and 2015 (93.56% to 89.52%). In South Korea, the incidence of THA in patients with ONFH (86%) is much higher than that of other surgical methods [12]. THA can effectively relieve joint pain, restore joint function, and improve the quality of life in patients during the collapse phase [13].

Individuals between the ages of 30 and 50 years have been identified as the most susceptible to ONFH [14]. In recent years, with the increasing retirement age in many countries, including China, the ability of patients undergoing THA to return to work (RTW) has become increasingly economically important. Although many patients are unlikely to choose THA for the sole purpose of improving their quality of work, research shows that employment is an important aspect of overall quality of life, especially in young, active patient groups [15]. The expectation for a quick and normal RTW after THA is rising among patients. Considering the need for a longer working life and patients' expectations regarding their abilities to perform activities postoperatively, RTW is considered an important marker of surgical success. Therefore, RTW or postoperative improvement after THA is a major concern for patients. However, studies on RTW in patients after THA in China and other regions of Asia have been less extensive. A detailed understanding of the timing and predictive factors of RTW after THA will help to guide the rapid recovery of patients undergoing THA.

This study aimed to evaluate the status of RTW in patients with ONFH who received THA and determine the predictive factors for successful RTW.

## Methods

### Setting

This retrospective study was approved by the Ethics Committee of Honghui Hospital affiliated with Xi'an Jiaotong University [grant numbers: 202204006]. Informed consent was obtained, from each participant. All subjects received underwent THA because of ONFH between January 2020 and December 2020 in the Department of Osteonecrosis and Joint Reconstruction of Xi'an Honghui Hospital.

### Participants

The inclusion criteria were as follows: (1) had a preoperative diagnosis of ONFH (Association Research Circulation Osseous [ARCO] [4, 16] stages III and IV); (2) worked 12 months preoperatively; (3) was aged ≤ 65 years at the time of THA; and (4) underwent primary unilateral THA.

Meanwhile, the exclusion criteria were as follows: (1) underwent bilateral THA or revision THA; (2) had malignancy, psychiatric illness, acute cardiovascular or cerebrovascular disease (multiple lacunar infarcts, myocardial infarction, sequelae of cerebral infarction, post pacemaker placement, etc.); (3) was considering prosthetic replacement of the affected limb on the other side or other surgical treatment within 1 year postoperatively; (4) was retired; and (5) was lost to follow-up.

Standardized surgical techniques and implants were used, and 20 consultant surgeons performed the arthroplasty in this study. Surgical techniques and implant use have been standardized. The postoperative rehabilitation measures were also standardized. At 1–2 days postoperatively, static contraction exercises of the quadriceps and passive straight leg raising and flexion hip exercises were performed with active mobilization of the ankle joint. At 3–7 days postoperatively, active ankle movement was continued. The patient was placed in a semi-sitting position either on or at the edge of the bed with the affected limb down and care taken to ensure that the flexion hip angle was not > 90°. After the patient was able to actively raise their legs and control the activities on the operated side, standing and walking with the protection of a walker and accompanying persons was performed. Eight to 14 days postoperatively, strengthening and physical recovery of the affected limb muscle was continued with straight leg raises and step-by-step ambulation activity with a walker. The flexion hip angle of the affected limb was not > 90° for 1 month postoperatively and, pillow protection between both knees in the lateral decubitus position was required. Squatting movements were gradually practiced after 3 months based on the patient’s rehabilitation.

### Study design and variables

The baseline characteristics of all patients were obtained from the hospital patient database, including sex, age, occupation, body mass index (BMI), preoperative conditions, underlying diseases, presence or absence of smoking, presence or absence of excessive drinking, American Society of Anesthesiologists’ patient profile and surgical risk score, preoperative Numerical Rating Scale (NRS) score [17], preoperative Harris hip score (HHS) [18], and contact details. Follow-up was conducted by video call, which was divided into two parts. The first component was comprised of questions on the patient’s RTW status (The indicator of RTW was the beginning of acquiring economic income through labor), the types of work that were performed preoperatively and postoperatively, length of time leaving work preoperatively, length of time returning to work postoperatively (from the end of surgery to complete RTW), change in the type of physical work that was performed preoperatively (less, same, or more), change in the length of work postoperatively (less, same, or more), and reasons for stopping work (e.g., retirement, hip problems, or other health problems).

Based on the International Classification of Occupations, two senior physicians assessed the patients’ work grades according to the national standard manual work intensity grading of the People’s Republic of China combined with patients’ work scenarios and joint workloads as follows: light work (such as staff or self-employed individuals), medium work (such as drivers or chefs), and heavy work (such as workers or farmers). Disagreements were resolved through mutual discussion.

The second part of the follow-up included the NRS and HHS scores. An HHS score of ≥ 90 was defined as excellent, 80–90 was defined as good, 70–80 was defined as general, and < 70 was defined as poor. The self-assessment of workability was defined as a patient’s (“current physical work ability” from the Work Ability Index [19] and was measured on a scale, from 0, “completely unable to work”, to 10, “normal ability to work”). The Likert scale satisfaction assessment was used to evaluate a patient’s ability to work after THA, and the participants selected any of the following answers: strongly disagree, disagree, neither agree nor disagree, agree, and strongly agree, with corresponding scores of 0–4, respectively.

### Statistical analysis

Descriptive and categorical data were collated into a Microsoft Excel spreadsheet, and descriptive statistics were used to obtain the counts and percentages. The qualitative content analysis was conducted through a systematic review of the free-text responses. For normally distributed variables, an independent samples t-test was used for two-group sample comparisons, and a one-way analysis of variance was performed to compare continuous variables with multiple groups. The rank sum test was used for continuous non-normally distributed variables, and the chi-square test was used for dichotomous variables. Using the receiver operating characteristic curve (ROC curve), predict and evaluate indicators with statistical differences to determine the predictive factors affecting RTW. All analyses were performed using the SPSS software (version 26.0; IBM, Armonk, New York, USA). Statistical significance was set at *P* < 0.05.

## Results

**Fig. 1:**
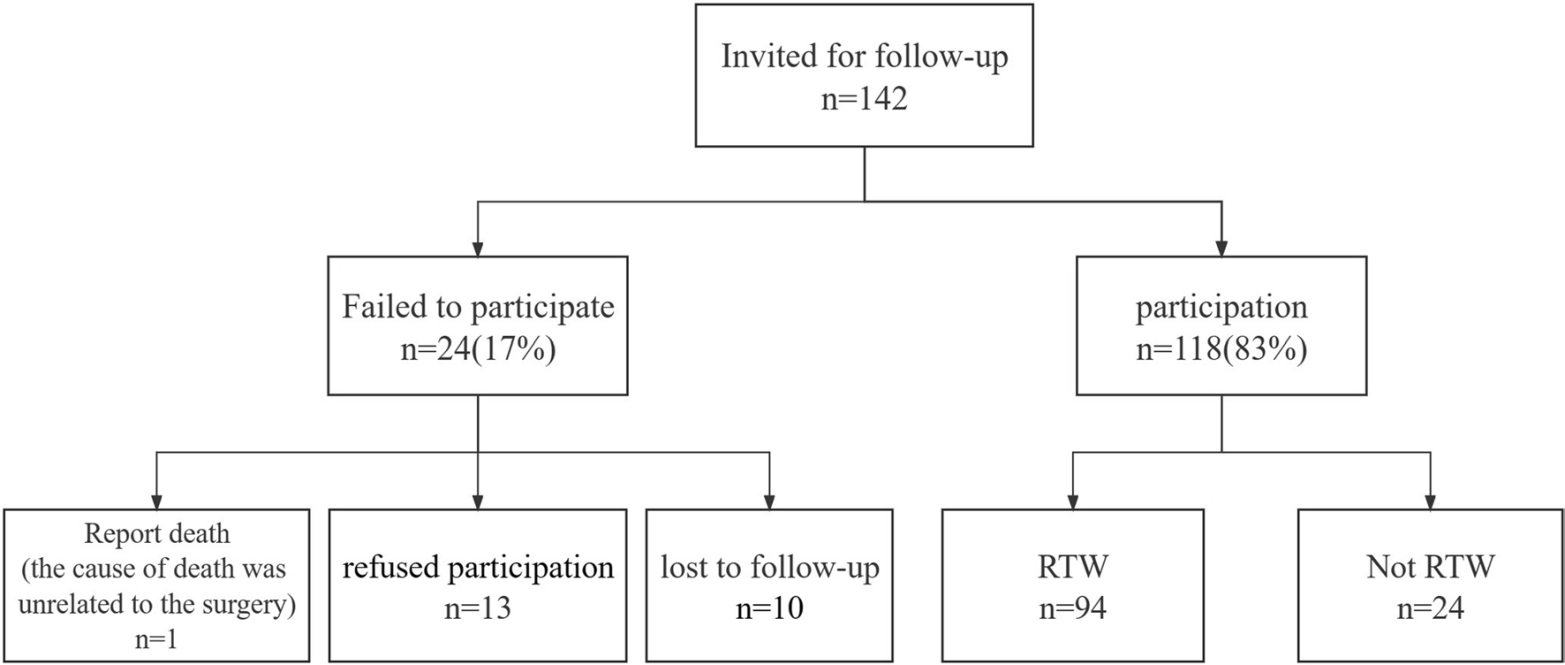
Flowchart of patient inclusion

A total of 142 patients met the inclusion criteria for this study, of whom 118 patients (83%) were followed up by telephone (n = 118; median age: 50 [interquartile range, 37–56; range, 20–64]; mean BMI: 24.09 [standard deviation, 2.83; range, 16.53–30.48]; and 78 patients [66%] were male). A flowchart of the patient inclusion results is provided in Fig. 1. Baseline characteristics and comparisons between the groups are presented in Table 1. Significant differences in smoking (*P* = 0.027 chi-square test), drinking (*P* = 0.002 chi-square test), and cardiovascular diseases (*P* = 0.012 chi-square test) were observed between the RTW and Non-RTW populations.

**Table 1:** Baseline characteristics of all patient who underwent THA and worked within 1 years prior to surgery (${\overline{\text{x}}}$ ± s)

Variable	RTW (n = 94)	Not RTW (n = 24)	All (n = 118)	*P* value
Age (years)	46.06 ± 11.34	50.79 ± 11.57	47.03 ± 11.50	0.967^a^
BMI (kg/m^2^)	24.28 ± 2.65	23.66 ± 3.48	24.16 ± 2.83	0.171^a^
	n (%)			
Sex: male	70 (75)	8 (33)	78 (66)	0.000^b**^
Type				
Traumatic	10 (10.6)	5 (20.8)	15 (12.7)	
non-traumatic	84 (89.4)	19 (79.2)	103 (87.3)	0.181^b^
ASA				
1 or 2	64 (68.1)	15 (62.5)	79 (66.9)	
3 or 4	30 (31.9)	9 (37.5)	39 (33.1)	0.604^b^
Smoking				
No	39 (41.5)	16 (66.7)	55 (46.6)	
Yes	55 (58.5)	8 (33.3)	63 (53.4)	0.027^b*^
Drinking				
No	37 (39.4)	18 (75.0)	55 (46.6)	
Yes	57 (60.6)	6 (25.0)	63 (53.4)	0.002^b**^
Hormonal history				
No	54 (57.4)	13 (54.2)	67 (56.8)	
Yes	40 (42.6)	11 (45.8)	51 (43.2)	0.772^b^
Diabetes	6 (6.4)	2 (8.3)	8 (6.8)	0.734^b^
Hypertension	26 (27.7)	9 (37.5)	35 (29.7)	0.346^b^
Respiratory Diseases	6 (6.4)	0 (0)	6 (5.1)	0.204^b^
Cardiovascular Diseases	9 (9.6)	7 (29.2)	16 (13.6)	0.012^b*^
Liver and kidney diseases	6 (6.4)	0 (0)	6 (5.1)	0.204^b^

*ASA* American Society of Anesthesiologists patient profile and surgical risk rating

^a^Independent sample t-test

^b^Chi-square test

*Significant difference (*P* < 0.05). **Extremely marked difference (*P* < 0.01)

Time to when preoperative work stopped and time to RTW.

**Fig. 2:**
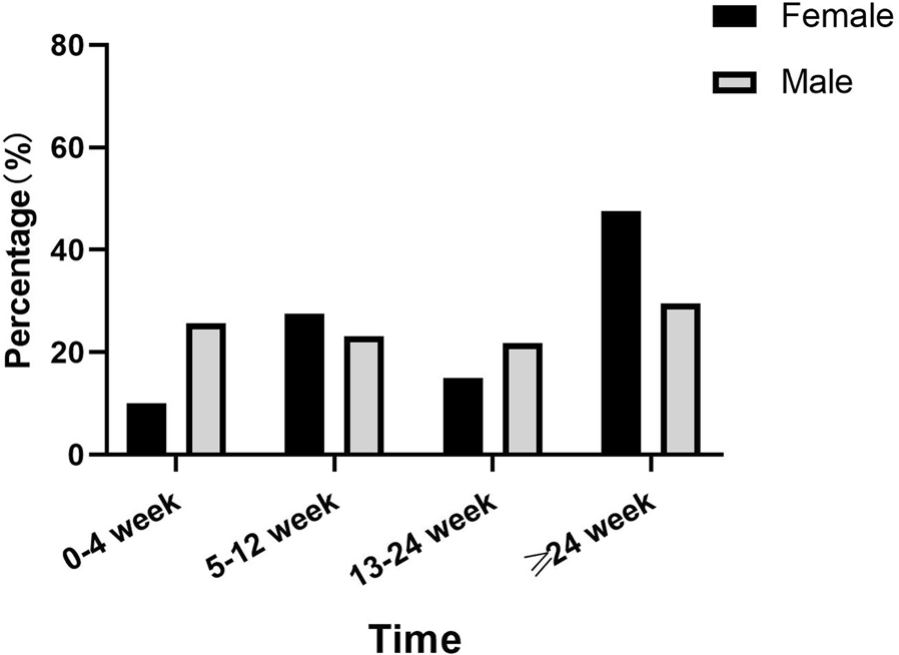
Distribution of the length of time when patients stopped work before undergoing total hip arthroplasty

The average length of time for the patients to stop working preoperatively was 20.7 weeks (SD, 15.3). Of the female patients, 19 (48%) stopped working preoperatively for > 24 weeks. Among the male patients, 20 (26%) stopped working within 4 weeks, 18 (23%) stopped at 5–12 weeks, 17 (22%) stopped at 13–24 weeks, and 23 (30%) stopped at > 24 weeks preoperatively (Fig. 2).

**Fig. 3:**
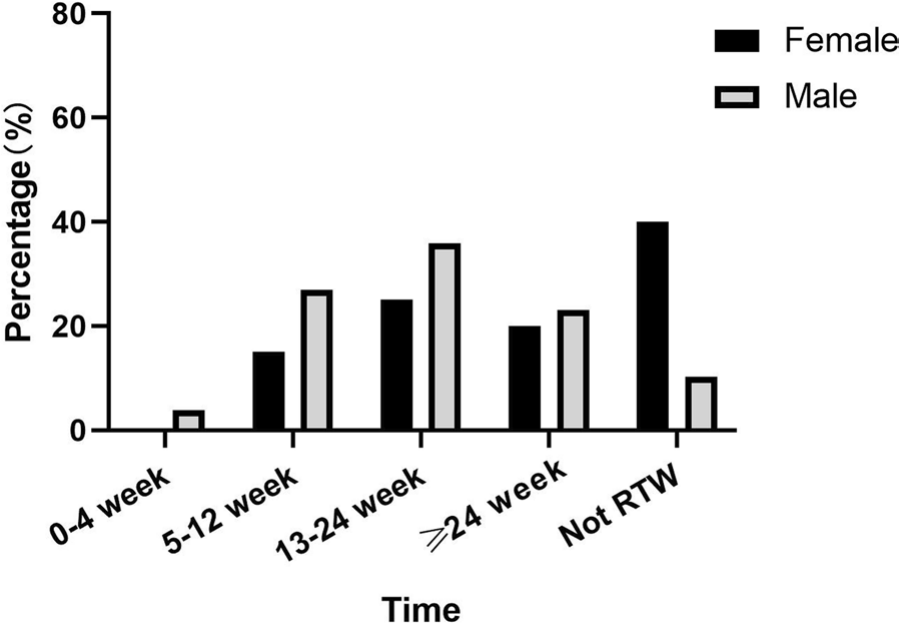
Distribution of the length of time when patients returned to work after undergoing total hip arthroplasty

In total, 94 patients returned to work after THA (24 women (60%) and 70 men (90%)) (Table 2). The mean duration to RTW was 21.0 weeks (SD, 11.4), including six (15%) women with a positive RTW status within 5–12 weeks, 10 (25%) within 13–24 weeks, and eight (20%) within > 24 weeks postoperatively. Additionally, three (4%) men had a positive RTW status < 4 weeks, 21 (27%) had 5–12 weeks, 28 (36%) had 13–24 weeks, and 18 (23%) had a positive RTW status after 24 weeks postoperatively (Fig. 2). The proportion of final positive RTW status was significantly higher in men than in women, and RTW was significantly faster in men than in women (P = 0.002, chi-square test) (Fig. 3 and Table 3).

**Table 2:** Status of patients returning to work

Variable	RTW	Not RTW	All	*P* value
	(n = 94)	(n = 24)	(n = 118)	
	n (%)			
Sex: male	70 (75)	8 (33)	78 (66)	0.000^a**^
Preoperative level of work				
Low	30 (32)	5 (21)	35 (30)	
Medium	30 (32)	10 (42)	40 (34)	
Heavy	34 (36)	9 (38)	43 (36)	0.512^a^
Postoperative level of work				
Low	40 (43)		40 (34)	
Medium	36 (38)		36 (31)	
Heavy	18 (19)		18 (15)	
Change in working level				
Reduce	25 (26.6)	24 (100)	49 (42)	
Equal	64 (68.1)	0 (0)	64 (54)	
Increase	5 (5.3)	0 (0)	5 (4)	0.000^a**^
Variation in the type of physical work				
Reduce	21 (22)	17 (71)	38 (32)	
Equal	41 (44)	5 (21)	46 (39)	
Increase	32 (34)	2 (8)	34 (29)	0.000^a**^
Change in working hours				
Reduce	14 (15)	8 (33)	22 (19)	
Equal	40 (43)	13 (54)	53 (45)	
Increase	40 (43)	3 (13)	43 (36)	0.012^a*^
Report of pain symptoms	17 (18)	10 (42)	27 (23)	0.014^a*^
Report of numbness symptoms	10 (11)	4 (17)	14 (12)	0.415^a^
Report of Poor activity symptoms	10 (11)	4 (17)	10 (8)	0.106^a^
Report of fatigue symptoms	15 (16)	6 (25)	21 (18)	0.301^a^
Pre-surgery: mean (sd)				
Harris score	49.21 ± 5.04	48.67 ± 4.73	49.10 ± 4.96	0.762^b^
NRS score	6.43 ± 0.99	6.13 ± 0.74	6.36 ± 0.95	0.139^b^
Post-surgery: mean (sd)				
Harris score	75.36 ± 5.26	71.08 ± 7.06	74.49 ± 5.90	0.006^b**^
NRS score	2.15 ± 0.95	2.67 ± 1.09	2.25 ± 1.00	0.039^b*^
Self-assessment of work ability	6.31 ± 1.32	4.88 ± 1.42	6.02 ± 1.46	0.000^b**^
Likert scale satisfaction assessment	3.13 ± 0.64	2.88 ± 0.80	3.08 ± 0.68	0.137^b^

*Significant difference (*P* < 0.05)

**Extremely marked difference (*P* < 0.01)

^a^Chi-square test

^b^Mann–Whitney U test

**Table 3:** Descriptive statistics for individuals by RTW status

	Not RTW	Post-surgery RTW by:			*P* value
	n = 24	0–12 week n = 30	13–24 week n = 38	≥ 24 week n = 26	
Age: mean (sd)	50.79 ± 11.57	46.30 ± 11.52	46.55 ± 10.49	45.08 ± 12.65	0.323^c^
BMI: mean (sd)	23.66 ± 3.48	24.83 ± 2.84	23.83 ± 2.35	24.32 ± 2.82	0.396^c^
	n (%)				
Sex: male	8 (33)	24 (80)	28 (74)	18 (69)	0.002^a**^
Smoking	8 (33)	15 (50)	25 (66)	15 (58)	0.087^a^
Drinking	6 (25)	18 (60)	23 (61)	16 (62)	0.021^a*^
Hormonal history	11 (46)	13 (43)	19 (50)	8 (31)	0.490^a^
Diabetes	2 (8)	3 (10)	1 (3)	2 (8)	0.647^a^
Hypertension	9 (38)	11 (37)	9 (24)	6 (23)	0.457^a^
Respiratory Diseases	0 (0)	2 (7)	3 (8)	1 (4)	0.543^a^
Cardiovascular Diseases	7 (29)	4 (13)	2 (5)	3 (12)	0.063^a^
Liver and kidney diseases	0 (0)	3 (10)	1 (3)	2 (8)	0.305^a^
Preoperative level of work					
Low	5 (21)	16 (53)	9 (24)	5 (39)	
Medium	10 (42)	7 (23)	13 (34)	10 (39)	
Heavy	9 (38)	7 (23)	16 (42)	11 (42)	0.045^b*^
Postoperative level of work					
Low		17 (57)	14 (37)	9 (35)	
Medium		8 (27)	13 (34)	15 (58)	
Heavy		5 (17)	11 (29)	2 (8)	0.047^a*^
Change in working level					
Reduce	24 (100)	3 (10)	10 (26)	12 (46)	
Equal	0 (0)	26 (86)	26 (68)	12 (46)	
Increase	0 (0)	1 (3)	2 (5)	2 (8)	0.000^b**^
Variation in the type of physical work					
Reduce	17 (71)	6 (20)	10 (26)	5 (19)	
Equal	5 (21)	15 (50)	15 (40)	11 (42)	
Increase	2 (8)	9 (30)	13 (34)	10 (39)	0.001^b**^
Change in working hours					
Reduce	8 (33)	5 (17)	6 (16)	3 (12)	
Equal	13 (54)	13 (43)	15 (40)	12 (46)	
Increase	3 (13)	12 (40)	17 (45)	11 (42)	0.031^b*^
Report of pain symptoms	10 (42)	5 (17)	9 (24)	3 (12)	0.061^a^
Report of numbness symptoms	4 (17)	3 (10)	5 (13)	2 (8)	0.772^a^
Report of Poor activity symptoms	4 (17)	1 (3)	4 (11)	1 (4)	0.259^a^
Report of fatigue symptoms	6 (25)	5 (17)	6 (16)	4 (15)	0.781^a^
Pre-surgery: mean (sd)					
Harris score	48.67 ± 4.73	50.17 ± 4.58	48.87 ± 5.10	48.62 ± 5.47	0.599^c^
NRS score	6.13 ± 0.74	6.50 ± 0.90	6.26 ± 0.89	6.58 ± 1.21	0.277^c^
Post-surgery: mean (sd)					
Harris score	71.08 ± 7.06	77.03 ± 4.45	73.74 ± 5.22	75.81 ± 5.65	0.001^c**^
NRS score	2.67 ± 1.09	1.93 ± 0.74	2.29 ± 1.18	2.19 ± 0.75	0.059^c^
Self-assessment of work ability	4.88 ± 1.42	6.67 ± 1.52	6.16 ± 1.29	6.12 ± 1.07	0.000^c**^
Likert scale satisfaction assessment	2.88 ± 0.80	3.20 ± 0.48	3.03 ± 0.79	3.19 ± 0.57	0.259^c^

0–4 week and 5–12 week data have been combined as only three individuals returned to work between 0 and 4 weeks for total hip arthroplasty

*Significant difference (*P* < 0.05)

**Extremely marked difference (*P* < 0.01)

^a^Chi-square test

^b^Kruskal–Wallis test

^c^F-test

### Changes in physical burden at work by the RTW or not status

Postoperatively, the patients with a positive RTW status had the highest percentage of engagement in light work (43%). Sixty-four (68.1%) patients with a positive RTW status had consistent preoperative and postoperative type of work. Significant differences were observed between the two groups in changes in working levels, variations in the types of physical work, and changes in working hours (*P* < 0.001 chi-square test; *P* < 0.001 chi-square test; *P* = 0.012 chi-square test). Postoperative pain, numbness, poor activity, and fatigue were the most frequent postoperative complications in the follow-up population, with the largest number (23%) of patients experiencing pain symptoms after THA and there was a statistical difference between the two groups (*P* = 0.014 chi-square test) (Table 2).

The patients with a positive RTW status had higher postoperative HHS scores [75.36 (SD 5.26) vs 71.08 (SD 7.06) (*P* = 0.006, Mann–Whitney U test)], lower postoperative NRS scores [2.15 (SD 0.95) vs 2.67 (SD 1.09) (*P* = 0.039, Mann–Whitney U test], and higher self-assessment of work ability [6.31 (SD 1.32) vs 4.88 (SD 1.42) (*P* < 0.000, Mann–Whitney U test)] than patients who had a negative RTW status. No other correlation scores were significantly associated with RTW (Table 2). Regarding the satisfaction evaluation, 21% of the non-RTW patients were dissatisfied with their work abilities, while 8.5% of the RTW patients were dissatisfied with their work abilities.

**Fig.4:**
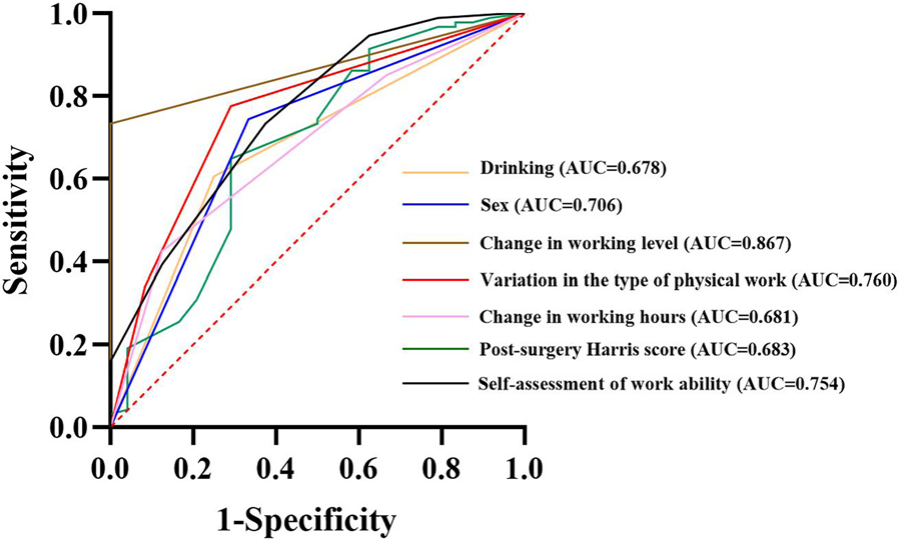
ROC curve for status of returning to work

Predict indicators with statistical differences using ROC curves, and the results show that drinking (*P* = 0.007, AUC = 0.678, 95% CI: 0.561–0.795), sex (*P* = 0.002, AUC = 0.706, 95% CI: 0.585–0.827), change in working level (*P* < 0.000, AUC = 0.867, 95% CI: 0.804–0.930), variation in the type of physical work (*P* < 0.000, AUC = 0.760, 95% CI: 0.652–0.868), change in working hours (*P* = 0.006, AUC = 0.681, 95%CI: 0.567–0.795), post-surgery Harris score (*P* = 0.006, AUC = 0.683, 95%CI: 0.554–0.812) and self-assessment of work ability (*P* < 0.000, AUC = 0.754, 95%CI: 0.644–0.865) can serve as a predictive factor (Fig. 4). However Cardiovascular Diseases(*P* = 0.140), smoking (*P* = 0.058), and post-surgery NRS score (*P* = 0.52) cannot be used as predictive factors for RTW.

### Changes in physical burden at work by RTW status

When patients with different RTW statuses were considered, the largest number of patients (38 (32%)) took 13–24 weeks to RTW. The highest percentage (42%) of non-RTW patients was preoperatively engaged in an intermediate level of work. The preoperative work level of patients who returned to work within 0–12 weeks was primarily low (53%), but patients who returned to work within 13–24 weeks and ≥ 24 weeks were primarily engaged in medium and heavy work (76% and 81%, respectively). However, for patients who achieved a positive postoperative RTW status, those who returned to work within 0–12 weeks and those who returned within 13–24 weeks reported the highest proportion of low work levels postoperatively (57% and 37%, respectively). Patients with an RTW status of ≥ 24 weeks were primarily engaged in medium work (58%). Significant differences in the preoperative and postoperative job types were noted between the different RTW populations (*P* = 0.045, Kruskal–Wallis test; *P* = 0.047, chi-square test) (Table 3).

Regarding variations in the types of physical work, 71% of the non-RTW patients had a reduction in the type of physical work, whereas 78% of the RTW population perceived the type of physical work to be the same or increased compared with that before surgery, with significant differences between the two groups (*P* = 0.001 Kruskal–Wallis test). Regarding changes in working hours, the RTW patients had a higher proportion and significantly different increases in working hours compared with those of the non-RTW patients (*P* = 0.031, Kruskal–Wallis test). Nevertheless, both groups experienced postoperative pain, numbness, poor activity, and fatigue (Table 3).

## Discussion

To the best of our knowledge, this is the first clinical study in China to focus on RTW status after THA in patients with ONFH. The results of this study might be extrapolated to the relevant situation in China and East Asian countries. There are a few literature reports on returning to work after THA, and these studies primarily occurred in European and American countries. Ryan et al. [20] conducted a follow-up study in the United States on patients who underwent THA and were under 60 years; the authors found that the majority of young, active patients who were employed before surgery were expected to RTW (90.4%). Khalid et al. [21] conducted a prospective study in the UK on the effects of patient intent and preoperative work status and found that patients who intended to RTW following THA had a postoperative RTW rate of 88.5%. Raul et al. [22] reported in a national cohort study in Finland that 94% of patients who underwent THA had a positive RTW status after 3 months (10 days to 1 year) of sick leave. Moreover, Eric's prospective research report showed an RTW rate of 74% after THA [23]. However, most of the previous reports were primarily focused on patients who underwent THA and did not pay closer attention to the population of patients with ONFH. Economic levels, healthcare systems, and cultures vary globally, as do the RTW rates. Moreover, it is important to consider Chinese population-specific data. Fortunately, although most of these studies have focused on European and American countries, the rate of RTW after THA in China (80%) is not different from that in countries such as Europe and the United States. The present study demonstrated that the majority of patients (women, 63%; men, 51%) had stopped working ≥ 3 months before THA because of the effect of ONFH. By contrast, 54% of patients required ≥ 3 months to RTW after THA (women, 45%; men, 59%). Other studies have reported that approximately half of the patients who underwent THA returned to work within 3 months postoperatively, with a higher proportion of patients who underwent THA returning to work within 1-month postoperatively [22, 24, 25]. However, in this study, the mean RTW status after THA was 21.0 weeks (SD, 11.4), which is longer for patients in China. Interestingly, during the telephone follow-up, most patients self-perceived that postoperative recovery was beneficial by extending the duration of recovery. Bardgett et al. [26] revealed that similar employee habit adaptation, staged RTW, and workload reduction contribute to RTW. These results suggest that reinforcement in terms of rehabilitation guidance and teaching after THA are necessary as well as more observations and research on when to RTW and a determination of a suitable time point for clinical guidance.

Regarding the level of work, 64 (68.1%) RTW patients had consistent preoperative and postoperative work types, 25 (26.6%) patients had decreased postoperative work types, and only five (5.3%) patients had increased postoperative work types. At different periods of RTW, we observed that the work status of patients with a time to RTW of 0–12 weeks remained consistent, while the proportion of patients with a time to RTW that was greater than 12 weeks with reduced work types increased. This is because RTW patients who returned to work within 0 to 12 weeks were more likely to have engaged in mild to moderate work before surgery, with lower work intensity and shorter postoperative recovery time to cope with the work environment. Sex, drinking, change in working level, variation in the type of physical work, change in working hours, and length of time to RTW were found as important factors in determining RTW status, which is consistent with the results of previous studies [27].

Interestingly, when considering the distribution of patients' occupations, female patients had the highest percentage (55%) of farmer occupations; male patients had the highest proportion of occupations as workers (32%), followed by farmers (19%). Male patients, who are the primary workforce of the family, mostly continue to engage in physical activity shortly after undergoing surgery, whereas female patients have the option of living at home for household chores postoperatively (11 patients), accounting for 69% of the female population who do not RTW postoperatively. Patients who RTW more quickly tend to report more pain, functional limitations, and work limitations than patients who RTW later in the postoperative period. Considering the crop harvest times for crops that are grown in northern China, we found a high incidence of THA (64%) after this period among patients who are farmers. With the increase in work intensity, the development of ONFH has accelerated, and patients are motivated to receive THA treatment.

Previous studies of patients who similarly underwent THA have demonstrated no significant correlation between preoperative scores and RTW status in this patient group [28]. Although most patients exhibit significant functional improvement after THA as measured by HHS and RNS, a subset of patients who do not RTW remains, with a higher proportion of those patients being women. Thus, the willingness to receive surgery, long hospitalization, postoperative analgesia of patients, and the presence of psychological changes may complicate RTW [28]. These scores may not report all functional variables that are important to the patient, although they are well-validated and widely used [29, 30]. Subsequent research should consider a design from the three aspects of physiology, psychology, and economy. However, comparing RTW data across countries because of different social work environments, ages at retirement, welfare policies and pension arrangements, and cultural attitudes at work is difficult [21, 22]. Predicting RTW is a multifactorial decision-making process involving social, identity, personality, and financial factors.

## Limitations

This study had some limitations. First, this is a single-center study, and the rate of returning to work are not comparable. Second, the sample size was limited, and some patients were lost to follow-up or refused follow-up, which affected the data integrity and may affect the accuracy of the ROC curve. Third, the patients who were included in this study were required to have experienced work status within 12 months preoperatively, and the resulting RTW rate might be relatively high. Fourth, HHS and NRS scores were used as measures of pain, function, and activity levels, and these ratings were patient reported by video, with testers failing to make finer objective measurements, which may have introduced bias into the data. Fifth, the willingness of the patients to work was not examined preoperatively, and lastly the work-related questionnaire that was used in this study was not validated; however, the responses were reported separately and were not used to calculate the scores.

## Conclusion

In this study, 80% of patients with ONFH who received THA achieved RTW status after surgery. Drinking, sex, change in working level, variation in the type of physical work, change in working hours, post-surgery Harris score and self-assessment of work ability can serve as predictive factors for RTW.

## Data Availability

Due to patient privacy and confidentiality considerations, the dataset that was generated and/or analyzed as part of the present study is not publicly available, but can be obtained from the corresponding authors upon reasonable request.
